# Vitamin D Receptor Gene Variants Susceptible to Osteoporosis in Arab Post-Menopausal Women

**DOI:** 10.3390/cimb43030094

**Published:** 2021-09-27

**Authors:** Mohammed. G. A. Ansari, Abdul Khader Mohammed, Kaiser A. Wani, Syed D. Hussain, Abdullah M. Alnaami, Saba Abdi, Naji J. Aljohani, Nasser M. Al-Daghri

**Affiliations:** 1 Chair for Biomarkers of Chronic Diseases, College of Science, King Saud University, Riyadh 11451, Saudi Arabia; mansari@ksu.edu.sa (M.G.A.A.); makhaderonline@gmail.com (A.K.M.); kwani@ksu.edu.sa (K.A.W.); shussain@ksu.edu.sa (S.D.H.); aalnaami@ksu.edu.sa (A.M.A.); najij@hotmail.com (N.J.A.); 2 Sharjah Institute for Medical Research, University of Sharjah, Sharjah 27272, United Arab Emirates; 3 Biochemistry Department, College of Science, King Saud University, Riyadh 11451, Saudi Arabia; sabdi@ksu.edu.sa; 4 Obesity, Endocrine, and Metabolic Center, King Fahad Medical City, Riyadh 59046, Saudi Arabia

**Keywords:** VDR gene polymorphism, Saudi women, bone mass density

## Abstract

Post-menopausal osteoporosis (PMO) is a multifactorial bone disorder in elderly women. Various vitamin D receptor (VDR) gene variants have been studied and associated with osteoporosis in other populations, but not in a homogenous Arab ethnic group. Herein, the current study explores the association between VDR polymorphisms and susceptibility to osteoporosis in Saudi postmenopausal women. In total, 600 Saudi postmenopausal women (N = 300 osteoporosis; N = 300 control) were genotyped for VDR gene variants (*rs7975232*, *rs1544410*, *rs731236*) using TaqMan^®^ SNP genotyping assays. Bone mineral density (BMD) for the lumbar spine and femur was assessed using dual-energy X-ray absorptiometry (DEXA). The heterozygous frequency distributions AC of *rs7975232*, CT of *rs1544410*, and AG of *rs731236* were significantly higher in the osteoporosis group than controls (*p* < 0.05). Heterozygous AC of *rs7975232* (1.6; 95% CI 1.1–2.3; *p* < 0.023), CT of *rs1544410* (1.6; 95% CI 1.1–2.4; *p* < 0.022), and AG of *rs731236* (1.6; 95% CI 1.1–2.4; *p* < 0.024) were significantly associated with increased risk of osteoporosis, independent of age and BMI. In conclusion, VDR gene variants *rs7975232*, *rs1544410*, *rs731236* had a significant effect on BMD and were associated with osteoporosis risk in Saudi postmenopausal women.

## 1. Introduction

Osteoporosis is a polygenic, multifactorial global health issue in the elderly population, characterized by low bone mineral density (BMD) and micro-architectural deterioration of bone tissue, with an impact on bone strength and skeletal fragility [1,2,3]. Generally, bone-remodeling processes (formation/resorption) are tightly maintained by the combined action of multiple genes and environmental influence, but this balance declines with age especially in elderly women, with a consequent decrease in bone calcium levels and increased susceptibility to postmenopausal osteoporosis (PMO) [4]. BMD is an excellent tool for assessing and predicting fracture risk and osteoporotic fractures [5]. In addition to environmental factors (physical activity, diet, etc.) findings from various twin and family studies reveal that BMD is under strong genetic influence, accounting for up to 50–80% of the inter-individual variance in bone mass [6,7,8]. Determining these gene variants related to low BMD might be clinically useful for individuals to curb or delay osteoporosis through early management and preventive monitoring. In addition, it will give a better molecular understanding of underpinning the disease pathogenesis.

In recent years, the traditional role of vitamin D (25(OH)D) in calcium homeostasis, bone tissue mineralization and remodeling via VDR have expanded through multiple studies that highlight the pleiotropic effects of 25(OH)D and its genetics in extra-skeletal functions and associations with various metabolic conditions [9,10,11]. VDRs are nuclear hormone receptors encoded by a gene located on chromosome 12q13.1 and consist of 14 exons capable of generating multiple tissue-specific transcripts [12]. Vitamin D mediates its calciotropic action after binding to VDR and modulates the transcription of several target genes that help in calcium uptake and/or bone formation [13]. Almost 200 single nucleotide polymorphisms (SNPs) have been detected in the VDR gene, but their impact on VDR protein function is largely unknown [14]. Different VDR gene variants have recently gained attention as many studies have confirmed their substantial associations with several diseases [15,16]. Among them were three VDR gene variants (*rs7975232*, *rs1544410*, and *rs731236*) located on the 3′-regulatory region of the VDR gene and are often marked as a single haplotype [14,17].

A recent epidemiologic study in Saudi Arabia illustrated that 34% of Saudi women (50–79 years of age) have osteoporosis [18]. Osteoporosis adversely affects the patients’ mobility, function, and quality of life, causing financial burden on their family in particular and on society in general [19,20,21]. Herein, we hypothesized that genetic variation in the VDR gene could be associated with risk of osteoporosis. Interestingly, no study on the association between VDR gene variants with BMD or osteoporosis risk was reported in Arab ethnicity; hence the rationale of this study was to investigate the influence of three VDR gene variants (*rs7975232*, *rs1544410*, and *rs731236*) on BMD and susceptibility to osteoporosis in Saudi postmenopausal women. While the selected gene variants have been most frequently studied with BMD and osteoporosis, the results remain non-conclusive [22], and observations from understudied ethnic groups such as the Arabian ethnicity may provide additional information.

## 2. Materials and Methods

### 2.1. Study Design and Participants

The current study is approved by the Ethics Committee of The College of Science, King Saud University (KSU), Riyadh, Kingdom of Saudi Arabia (KSA), No 8/25/36516, and all methods/protocols were carried out in accordance with relevant guidelines and regulations and abide by the principle of the Declaration of Helsinki.

The study participants include 600 Saudi post-menopausal women (300 osteoporosis and 300 control) recruited from different primary health care centers (PHCCs) in Riyadh, KSA. Prior to study enrolment, written informed consent was obtained from each participant. Demographic information and present and past medical history were gained through a general questionnaire.

### 2.2. Exclusion Criteria

Subjects with anti-osteoporosis treatment history or on medications known to interfere with bone metabolism and documented bilateral oophorectomy, hypogonadism, hypothyroidism, malignancies, hereditary bone disease or endocrine, cardiac and lung disease were excluded from the study. The shortlisted subjects underwent a medical examination and in addition, overnight fasting blood was withdrawn to determine serological parameters (glucose, albumin, phosphorous, calcium) related to liver and renal function. Participants who received normal outcomes were deemed eligible for this study.

### 2.3. Anthropometry and Blood Collection

Participants requested to visit their concerned PHCCs after overnight fasting (>10 h). Anthropometry variables include waist circumference, hip circumference, waist to hip ratio (WHR), body mass index (BMI) (kg/m^2^), systolic and diastolic blood pressure were measured. Peripheral blood for DNA extraction was collected in anticoagulant tubes (EDTA), whereas blood for serum was obtained in non-anticoagulant tubes. The isolated serum is stored at −80 °C at the Chair for Biomarkers of Chronic Diseases (CBCD) in KSU, Riyadh, KSA, until further use.

### 2.4. Biochemical Analysis

All analyses were performed at the CBCD laboratory, KSU, Riyadh, KSA. Lipid profile and fasting glucose levels were measured by the chemical analyzer (Konelab, Espoo, Finland). Serum 25(OH)D was measured with a Roche Elecsys modular analytics Cobas e411 using an electro-chemiluminescence immunoassay (Roche Diagnostics, GmbH, Mannheim, Germany). CBCD laboratory is participating in the Vitamin D External Quality Assessment Scheme (DEQAS) and quality assurance standards are according to ISO-17025. Vitamin D binding protein (VDBP), sclerostin (SOST) and all the other biochemical parameters were measured by following the protocol and instructions mentioned in our previous paper [23].

### 2.5. Bone Mass Density (BMD) Measurement

BMD (g/cm^2^) of lumbar vertebrae (L1–L4) and dual femur was assessed for all the selected subjects by using Prodigy-(GE Healthcare, Chicago, IL, USA), a dual-energy *x*-ray absorptiometry (DXA), and T-scores recorded in accordance with World Health Organization (WHO) guidelines. Based on these criteria, women with T-Scores higher than −1.0 were considered normal and below −1.0 were considered low BMD group. The detailed protocol was mentioned in our previous work [4,24].

### 2.6. VDR Genotyping

Genomic DNA was isolated from blood using the illustra^TM^ blood genomicPrep Mini Spin Kit (GE Healthcare, Chicago, IL, USA) and Nanodrop spectrophotometer (ND-1000, Nanodrop Technologies, Wilmington, DE, USA) quantified the DNA concentration of purified DNA. The three VDR SNPs—*rs7975232*, *rs1544410*, and *rs731236* were evaluated by allelic discrimination using real-time polymerase chain reaction (PCR) with predesigned TaqMan genotyping assays (Applied Biosystems, Foster City, CA, USA) in 10 µL reactions. Details related to TaqMan assay IDs and context sequence (primers and probes) were presented previously [23]. The PCR reactions were carried out in 96-well PCR plates using a Biorad CFX96 real-time PCR detection system (Bio-Rad, Milan, Italy). DNA amplification and detection were performed as follows: 95 °C for 10 min, followed by 45 cycles of 94 °C for 15 s and 60 °C for 1 min. Fluorescence detection occurred at 60 °C. Post PCR allelic discrimination was analyzed using Bio-Rad CFX manager software. Genotype assignment was determined by plotting relative fluorescent units (RFU) for one fluorophore (allele 1 on the *x*-axis) against the RFU for another fluorophore (allele 2 on the *y*-axis) on the allelic discrimination. All PCR reactions were set up in a dedicated PCR area with dedicated PCR pipettes and reagents. Genotype assignment was achieved by following the methods described in our previous paper [23] using the Bio-Rad CFX Manager software.

### 2.7. Statistical Analysis

Statistical calculations were performed using the Statistical Package for the Social Sciences for Windows (SPSS version 22.0, Chicago, IL, USA). Quantitative normal variables were expressed as mean ± standard deviation (SD), and the statistical mean differences between normal and PMO subjects were tested using an independent sample *t*-test. Quantitative non-normal variables were expressed as medians (Q1–Q3), and the statistical differences between normal and PMO subjects were tested using Mann–Whitney U test. Furthermore, analysis of covariance (ANCOVA) was used to adjust for age, BMI, age of menarche and menopause. Hardy–Weinberg equilibrium (HWE) tests were applied to assess the predicted genotype frequencies of the VDR gene variants. Allele and genotype were tested for risk of osteoporosis for each SNP using logistic regression, presented as odds ratio (OR) with 95% confidence interval (CI 95%). The common allele and genotype were used as the reference. Significance was set at *p* <  0.05.

## 3. Results

Table 1 shows the anthropometric and biochemical profiles of the study participants. The mean age of osteoporosis subjects (57.8 years ± 7.9) was significantly higher than control subjects (53.9 ± 6.1) (*p* < 0.01). Mean body mass index (BMI) was significantly lower in subjects with osteoporosis as compared to controls (31.5 kg/m^2^ ± 6.3 versus 34.3 kg/m^2^ ± 5.9; *p* < 0.01). Duration of menopause was significantly higher in the osteoporosis group than controls [median 8.0 years (4.0–15.0) versus 4.0 (2.0–8.0); *p* < 0.01). No differences in the biochemical profile of both groups were observed, except for triglycerides (TG) which were significantly lower in the osteoporosis group than controls (median 1.50 mmol/L (1.17–2.02) versus 1.61 mmol/L (1.25–2.27); *p* < 0.03).

Table 2 shows the association between VDR SNPs and osteoporosis. The risk of osteoporosis increased by 44% among subjects with the A/C genotype of *rs7975232* compared to the A/A genotype (*p* < 0.05). This risk further increased to 56% after adjusting for age and BMI (*p* < 0.05). Furthermore, subjects in *rs1544410* with the C/T genotype were 60% more likely to develop osteoporosis as compared to subjects with the C/C genotype (*p* < 0.05), independent of age and BMI. Moreover, for *rs731236*, odds of osteoporosis were greater in subjects with G/G and A/G genotypes with 68% and 44%, respectively, as compared to subjects with A/A genotype (*p* < 0.05). The risk of osteoporosis increased by 34% in subjects having G allele as compared to A allele (*p* < 0.05), but this significance was lost after adjusting for age and BMI.

The association analysis between the *rs1544410* genotypes, bone markers and other circulatory parameters are shown in Table 3, with minor GG and heterozygous AG genotype of *rs731236* polymorphism having significantly higher levels of sclerostin compared to major AA genotypes. Interestingly, these GG and AG genotypes were associated with decreased BMD at different sites. The correlations between *rs7975232* genotypes and various serological parameters were assessed using comparative analysis methods (Supplementary Table S1). Among all the circulatory parameters, no association has been identified with any of the studied genotypes. Bone turnover markers and other circulatory parameters do not exhibit any relationship with *rs1544410* genotypes in either of the studied groups (Supplementary Table S2). The association analysis between the *rs1544410* genotypes and bone markers and other circulatory parameters. It is observed that the bone turnover markers and other circulatory parameters do not exhibit any relationship with *rs1544410* genotypes in either of the studied groups.

## 4. Discussion

Epidemiological data revealed that osteoporosis is more prevalent in the elderly population, particularly in postmenopausal women. In addition to various other factors, BMD is strongly regulated by multiple genes. Gene polymorphisms in the VDR gene locus are known to be correlated with bone mass, hence its genotyping could be used to evaluate osteoporosis susceptibility. The VDR SNPs that are found strongly associated with BMD or PMO may serve as genetic markers for efficient screening and timely detection of the disease. Early diagnosis of PMO can help in better management of the disease as well as in slowing down its progress. In the current study, we assessed the role of three VDR gene variants (*rs7975232*, *rs1544410*, and *rs731236*) as the genetic determinant of BMD and osteoporosis in postmenopausal Saudis as polymorphism in this nuclear receptor gene plays a crucial role in osteoporosis pathogenesis. Results herein indicate that *rs7975232* and *rs731236* variants in the VDR gene are significantly associated with an increased risk of osteoporosis. Notably, *rs731236* variants are associated with significantly increased levels of serum sclerostin.

Vitamin D plays a crucial role in intestinal calcium absorption and bone metabolism; therefore, it is considered an important determinant of BMD. The active form of vitamin D, 1,25 (OH)2D3, binds with the cytosolic/nuclear VDR which then heterodimerizes with retinoid X receptor (RXR). The heterodimer thus formed, binds to vitamin D response elements (VDREs) and regulates transcription of target genes [25]. VDR modulates the transcription of various genes including osteocalcin and calcium-binding proteins which are involved in calcium homeostasis and bone formation [26,27,28]. The SNPs in VDR influence the expression and function of the VDR protein, which in turn affects the risk of low BMD and osteoporosis [28]. Morrison et al. were the first to document the relationship between the VDR gene variants and BMD at the spine and femur in Caucasian women [29,30]. Subsequently, in different ethnicities, extensive research has been carried out that reports an association between the allelic variants in the VDR gene and BMD [30,31,32,33]. However, the results have been inconsistent and inconclusive; the discrepancies between studies among diverse ethnic groups might be due to the differences in genetic backgrounds, allelic frequencies, and gene-environment or gene-gene interactions [13,31]. The most commonly studied VDR polymorphisms include *rs7975232*, *rs1544410* and *rs731236* located at the 3′ untranslated region of the gene, a region involved in the regulation of gene expression through modulation of mRNA stability [14,34].

SNP *rs731236*, located in exon 9 at the 3′ end of the VDR gene, results in a synonymous change and has been proved to affect mRNA stability [35,36]. The current study in agreement with previous studies by Marozik et al. and Lijuan Fu et al, confirms the occurrence of a significant association between VDR *rs731236* and risks of PMO [37,38]. The SNP *rs1544410* located in intron 8 at the 3′ end of the VDR gene is also known to be involved in the regulation of mRNA stability and has been associated with the increased risk of developing PMO in Caucasians [38]. A recent meta-analysis performed by Long-Liao et al., concluded that VDR SNP *rs1544410* has an association with increased risk of developing PMO among Caucasians but not among Asians [39]. Our study found the role of *rs1544410* (C/T) in the risk of PMO and thus verifies the results of the study of Long-Liao et al. as [39] and others [37,38], whereas the outcome of several other studies did not observe a significant association between VDR SNP *rs1544410* and risk of PMO [40,41,42]. The SNP *rs7975232* is located in the 3′-regulatory region of the VDR gene. The latest study reported an association of *rs7975232* homozygous genotype with osteoporosis risk in Egyptian women [43]. This contrasts our study which found heterozygous genotype (AC) of *rs7975232* to be associated with increased odds of having PMO as compared to the homozygous genotype (AA). Altogether, the results of our study on the role of three common VDR gene polymorphisms in the risk of PMO finds support in a meta-analysis by Yadav et al. who reported the association in the Caucasian population [44], while *rs731236* increased the risk of PMO whereas the other studied VDR SNPs (*rs1544410* and *rs7975232*) show no relation.

An interesting finding in the current study is the association of *rs731236* with serum sclerostin levels. Sclerostin is secreted by mature osteocytes during the completion of osteon formation [45]. It is a negative regulator of bone formation and a positive regulator of bone resorption [46]. Sclerostin acts as the inhibitor of the Wnt signaling pathway and thereby inhibits bone formation [45]. Mutations in the sclerostin coding gene cause sclerosteosis with low SOST levels and increased bone formation [40]. In our study, we found that minor GG and heterozygous AG genotype of *rs731236* polymorphism showed significantly higher levels of sclerostin compared to major AA genotype. Interestingly, these GG and AG genotypes were associated with decreased BMD at different sites.

It has been proposed that elevated TG levels play a crucial role in the overall enhancement of bone quality by creating by producing a layer between collagen fibers and mineral crystal [47]. We found a significant association between TG and BMD; we have previously demonstrated similar results where hypertriglyceridemia increased significantly with the increase in T-score tertiles [24]. Outcomes from the Dragojevic et al. study show similar results, which demonstrate that bone tissues in subjects with osteoporosis exhibit lower osteoblastogenesis, higher osteoclastogenesis and lower TG metabolism compared to their healthy counterparts [48].

The authors acknowledge some limitations. Large-scale studies with bigger sample sizes are warranted. As the subjects of our interest are selected only from Arab ethnicity, the findings of this study may have limited relevance to other populations. However, to the best of our knowledge, this study is the first of its kind in focusing on VDR polymorphism and osteoporosis in post-menopausal women of Arab ethnicity, particularly in Saudi subjects.

## 5. Conclusions

In conclusion, VDR gene variants (*rs7975232, rs1544410*, and *rs731236*) are associated with increased risk of osteoporosis and decreased BMD among Saudi Arabian post-menopausal women. The significant risk for PMO in VDR SNP *rs1544410* is similar to those found in Caucasian ethnicity. These SNPs might be useful genetic markers for the screening of PMO and may serve as a marker for early identification of Arab patients at high risk and perform the necessary preventive measures to avoid complications. Larger studies using the same ethnic group may serve to reinforce present findings. Other associations elicited in the present study such as those of TG and BMD, as well as *rs731236* with serum sclerostin levels are worthy of further exploration.

## Figures and Tables

**Table 1:** Anthropometry and clinical characteristics of subjects.

Parameters	Control N = 300	Osteoporosis N = 300	*p*-Value	*p*-Value *
**General characteristics**				
Age (Year)	53.9 ± 6.1	57.8 ± 7.9	<0.001	
BMI (kg/m^2^)	34.3 ± 5.9	31.5 ± 6.3	<0.001	
Menarche age (year)	13.1 ± 1.3	13.2 ± 1.6	0.74	0.70
Menopause status #	4.0 (2.0–8.0)	8.0 (4.0–15.0)	<0.001	
WHR	0.91 ± 0.10	0.92 ± 0.10	0.62	0.77
Systolic BP	126.6 ± 17.5	127.9 ± 18.8	0.40	0.51
Diastolic BP	76.3 ± 11.4	75.7 ± 10.7	0.50	0.89
Glucose (mmol/L)	7.8 ± 3.2	7.6 ± 3.3	0.57	0.88
Total Cholesterol (mmol/L)	4.97 ± 1.06	5.05 ± 1.06	0.36	0.43
HDL-cholesterol (mmol/L)	1.12 ± 0.3	1.16 ± 0.4	0.16	0.58
Triglycerides (mmol/L) #	1.61 (1.25–2.27)	1.50 (1.17–2.02)	0.03	0.07
25(OH)D (nmol/L) #	61.1 (37.8–84.5)	66.3 (39.1–93.0)	0.20	0.96
Vitamin D binding protein #	14.1 (5.9–53.8)	18.6 (5.9–70.1)	0.51	0.1
**T-Score and BMD**				
T-score AP spine #	−0.31± 0.83	−2.69 ± 0.67	<0.001	<0.001
T-score dual femur	0.44 ± 1.0	−1.08 ± 0.9	<0.001	<0.001
BMD spine	1.15 ± 0.13	0.86 ± 0.08	<0.001	<0.001
BMD dual femur left (g/mm^2^)	1.15 ± 0.14	0.85 ± 0.09	<0.001	<0.001
BMD dual femur right (g/mm^2^)	1.03 ± 0.12	0.84 ± 0.12	<0.001	<0.001
Average BMD	1.02 ± 0.12	0.84 ± 0.12	<0.001	<0.001
**Bone turnover markers**				
PTH (pg/mL) #	12.3 (7.5–21.0)	15.4 (7.8–34.9)	0.10	0.39
OPG (pg/mL) #	752 (523–995)	853 (617–1150)	0.04	0.48
OPN (ng/mL) #	2.3 (1.2–3.3)	2.7 (1.4–4.1)	0.11	0.48
SOST(pg/mL) #	1393 (647–2353)	1630 (939–2359)	0.23	63
FGF23(pg/mL) #	71.7 (42.5–82.0)	74.1 (43.6–82.4)	0.72	0.29
RANKL (pg/mL) #	33.1(18.5–61.0)	28.8 (21.7–48.3)	0.50	0.37
Osteocalcin (ng/mL) #	8.89 (3.2–13.13)	9.16 (3.45–14.19)	0.58	0.60
**Inflammatory markers**				
IL-4 (pg/mL) #	7.0 (3.9–10.3)	4.4 (2.5–9.3)	0.01	0.03
IL-1β (pg/mL) #	1.7 (0.3–2.7)	1.6 (0.3–2.6)	0.38	0.24
Leptin (ng/mL) #	16.1 (7.3–33.1)	20.2 (8.3–34.2)	0.32	0.11
TGF-β (pg/mL) #	40,264 (31,466–52,241)	37,583 (18,102–48,562)	0.048	0.33

Note: Data presented as mean ± SD and median (1st–3rd) percentiles for normal and non-normal variables (#). *p*-values were obtained from independent sample *t*-test and Mann–Whitney U test for normal and non-normal variables, respectively. * indicates *p*-values adjusted for age, BMI, age of menarche and menopause. WHR: waist-hip ratio; BP: blood pressure; PTH: parathyroid hormone; OPG: osteoprotegerin; OPN: osteopontin; SOST: sclerostin; FGF: fibroblast growth factor; RANKL: receptor activator of the nuclear factor-kappa-Β ligand; IL: interleukin; TGF: transforming growth factor. *p*-value < 0.05 was considered significant.

**Table 2:** Allelic frequency distribution of VDR gene variants.

Parameters	All	Control	Osteoporosis	Unadjusted		Adjusted	
				OR (95% CI)	*p*-Value	OR (95% CI)	* *p*-Value
*rs7975232*							
C/C	83 (13.8)	51 (17.0)	32 (10.7)	0.70 (0.42–1.16)	0.17	0.67 (0.38–1.19)	0.18
A/C	245 (40.8)	107 (35.7)	138 (46.0)	1.44 (1.01–2.05)	0.04	1.50 (1.01–2.25)	0.05
A/A	254 (42.3)	134 (44.7)	120 (40.0)	1		1	
A	753 (64.7)	375 (64.2)	378 (65.2)	1		1	
C	411 (35.3)	209 (35.8)	202 (34.8)	0.96 (0.75–1.22)	0.73	0.95 (0.73–1.25)	0.74
*rs1544410*							
T/T	124 (20.7)	63 (21.0)	61 (20.3)	1.17 (0.74–1.84)	0.5	1.19 (0.71–2.00)	0.59
C/T	276 (46.0)	128 (42.7)	148 (49.3)	1.39 (0.97–2.03)	0.08	1.65 (1.09–2.50)	0.02
C/C	190 (31.7)	104 (34.7)	86 (28.7)	1		1	
C	656 (55.6)	336 (56.9)	320 (54.2)	1		1	
T	524 (44.4)	254 (43.1)	270 (45.8)	1.12 (0.89–1.40)	0.35	1.14 (0.88–1.48)	0.31
*rs731236*							
G/G	119 (19.8)	52 (17.3)	67 (22.3)	1.68 (1.07–2.66)	0.02	1.49 (0.88–2.52)	0.14
A/G	265 (44.2)	126 (42.0)	139 (46.3)	1.44 (0.99–2.1)	0.05	1.57 (1.03–2.37)	0.03
A/A	203 (33.8)	115 (38.3)	88 (29.3)	1		1	
A	671 (57.2)	356 (60.8)	315 (53.6)	1		1	
G	503 (42.8)	230 (39.2)	273 (46.4)	1.34 (1.06–1.69)	0.01	1.28 (0.98–1.66)	0.07

Note: Data presented N (%). *p*-value significant at 0.05 and 0.01 level. Unadjusted ORs and *p*-values are obtained from binary logistic regression. Adjusted ORs and *p*-values are obtained from multinomial logistic regression. * *p*-value adjusted for age, BMI, age of menarche and menopause.

**Table 3:** Associations between *rs731236* genotypes and various other parameters.

rs731236	GG			AG			AA		
	Control	PMO	*p*-Value	Control	PMO	*p*-Value	Control	PMO	*p*-Value
**General characteristics**									
Age (year)	53.6 ± 6.9	59.5 ± 8.7	<0.01	53.5 ± 5.4	56.6 ± 7.2	0.00	54.4 ± 6.6	57.8 ± 8.2	<0.01
BMI (kg/m^2^)	33.4 ± 6.3	30.5 ± 7.2	0.03	34.5 ± 5.8	31.7 ± 5.8	0.00	34.7 ± 6.0	31.8 ± 6.3	<0.01
Menarche age (year) #	4.0 (2.0–6.0)	8.0 (4.0–15.0)	<0.01	4.0 (2.0–7.0)	8.0 (4.0–13.0)	0.00	5.0 (3.0–8.0)	7.0 (4.0–17.0)	<0.01
WHR	0.9 ± 0.1	0.9 ± 0.1	0.21	0.9 ± 0.1	0.9 ± 0.1	0.54	0.9 ± 0.1	0.9 ± 0.1	0.55
Systolic BP	122.4 ± 17.5	130.0 ± 20.3	0.04	127.7 ± 17.9	128.2 ± 19.0	0.86	127.6 ± 17.2	125.7 ± 17.1	0.45
Diastolic BP	75.7 ± 9.8	74.7 ± 10.7	0.63	76.1 ± 11.6	76.5 ± 11.1	0.83	77.0 ± 12.0	75.0 ± 9.9	0.23
Glucose (mmol/L)	7.2 ± 2.7	7.7 ± 3.5	0.38	8.2 ± 3.5	7.6 ± 3.3	0.16	7.5 ± 3.1	7.6 ± 3.1	0.82
T.Chol (mmol/L)	4.9 ± 1.0	4.8 ± 1.0	0.57	5.0 ± 1.0	5.1 ± 1.1	0.36	4.9 ± 1.1	5.1 ± 1.1	0.10
HDL-Chol (mmol/L)	1.1 ± 0.4	1.1 ± 0.4	0.90	1.1 ± 0.3	1.2 ± 0.4	0.20	1.1 ± 0.3	1.1 ± 0.3	0.42
TG (mmol/L) #	1.6 (1.1–2.1)	1.7 (1.2–2.1)	0.73	1.7 (1.3–2.3)	1.5 (1.2–2.1)	0.02	1.6 (1.2–2.2)	1.5 (1.2–2.0)	0.44
25(OH)D (nmol/L) #	67 (36–95)	64 (40–94)	0.92	57 (35–81)	67 (40–87)	0.05	62 (45–81)	67 (34–99)	0.91
VDBP #	7.6 (5.9–40.3)	16 (6.2–63.7)	0.27	19 (6.0–60.8)	24 (5.2–125)	0.78	13 (4.1–53)	10 (5.9–37.3)	0.79
**BMD**									
BMD spine	1.1 ± 0.1	0.8 ± 0.1	<0.01	1.2 ± 0.1	0.9 ± 0.1	0.00	1.2 ± 0.1	0.9 ± 0.1	<0.01
BMD DF left	1.0 ± 0.1	0.8 ± 0.2	<0.01	1.0 ± 0.1	0.9 ± 0.1	0.00	1.1 ± 0.1	0.8 ± 0.1	<0.01
BMD DF right	1.0 ± 0.1	0.8 ± 0.2	<0.01	1.0 ± 0.1	0.9 ± 0.1	0.00	1.0 ± 0.1	0.8 ± 0.1	0.00
**Bone turnover markers**									
PTH (pg/mL) #	14 (10–24)	16 (9–48)	0.54	13 (7–22)	15 (7–36)	0.71	11 (7–20)	15 (10–289)	0.07
OPG (pg/mL) #	752 (549–1168)	922 (669–1163)	0.29	783 (608–946)	844 (599–1072)	0.31	673 (465–1031)	806 (580–1206)	0.18
OPN (pg/mL) #	2843 (1520–5705)	2552 (1242–4098)	0.31	2218 (1217–3371)	2643 (1635–3856)	0.31	2311 (863–3064)	2902 (1462–3820)	0.05
SOST (pg/mL) #	1823 (695–2840)	1540 (674–2680)	0.64	1705 (788–2576)	1668 (1016–2442)	0.94	1021 (375–1585)	1615 (1086–2295)	0.04
FGF23 (pg/mL) #	73 (50–83)	73 (44–81)	0.70	73 (41–84)	75 (44–82)	0.80	68 (43–80)	73 (41–82)	0.89
Osteocalcin (ng/mL) #	11 (5.2–14.7)	8.8 (3.8–12.7)	0.30	8.5 (3.1–12.9)	10 (5.0–14.3)	0.11	8.8 (2.1–14.5)	8.4 (2.7–14.2)	0.84

Note: Data presented as mean ± SD for normal variables while median (Q1–Q3) for non-normal variables; # indicates non-normal variables. *p*-value < 0.05 considered significant; *p*-values are obtained from independent sample *t*-test and Mann–Whitney U test for normal and non-normal variables respectively. WHR: waist-hip ratio; BP: blood pressure; T. Chol: total cholesterol; TG: triglycerides; VDBP: vitamin D binding protein; DF: dual femur; PTH: parathyroid hormone; OPG: osteoprotegerin; OPN: osteopontin; SOST: sclerostin; FGF: fibroblast growth factor.

## Data Availability

The data presented in this study are available on request from the corresponding author.

## Supplementary Materials

The following are available online at https://www.mdpi.com/article/10.3390/cimb43030094/s1, Table S1: Association between *rs7975232* genotypes and various other parameters, Table S2: Association between *rs154440* genotypes and various other parameters.
