# Supplementary material for: Vitamin D Receptor Gene Variants Susceptible to Osteoporosis in Arab Post-Menopausal Women
Source: Curr Issues Mol Biol. 2021 Sep 27;43(3):1325–34. doi: 10.3390/cimb43030094 (PMC8929051; doi:10.3390/cimb43030094)
Supplement: Supplementary file 1 [file cimb-43-00094-s001.zip › cimb-1370318-supplementary.pdf]

Supplementary Table S1. Association between *rs7975232* genotypes and various other parameters

rs7975232	CC			AC			AA		
	Control	PMO	P-values	Control	PMO	P-values	Control	PMO	P-values
				**General Characteristics**					
Age (year)	53.7 ± 6.3	58.3 ± 9.0	0.01	54.0 ± 6.2	56.9 ± 7.6	<0.01	53.8 ± 6.1	58.4 ± 8.0	<0.01
BMI (kg/m^2)	34.1 ± 6.5	32.0 ± 5.9	0.16	34.6 ± 5.3	31.0 ± 5.7	<0.01	34.3 ± 6.3	32.0 ± 7.1	<0.01
Menarche (yr)#	5 (4 - 9)	7 (4 - 15)	0.11	4 (3 - 8)	8.0 (4.0 - 14.0)	<0.01	4.0 (2.0 - 6.0)	9.0 (4.0 - 15.0)	<0.01
WHR	0.9 ± 0.1	0.9 ± 0.1	0.21	0.9 ± 0.1	0.9 ± 0.1	0.54	0.9 ± 0.1	0.9 ± 0.1	0.55
Systolic BP	126.6 ± 15.4	124.6 ± 15.4	0.58	125.8 ± 17.0	126.7 ± 19.7	0.71	127.4 ± 18.9	129.9 ± 18.7	0.31
Diastolic BP	78.0 ± 11.5	75.5 ± 10.2	0.35	74.9 ± 11.8	75.7 ± 11.4	0.65	76.8 ± 11.2	75.5 ± 10.1	0.36
Glucose (mmol/l)	7.3 ± 3.0	7.0 ± 2.0	0.70	8.1 ± 3.5	7.7 ± 3.5	0.46	7.7 ± 3.1	7.7 ± 3.3	0.84
Chol (mmol/l)	4.8 ± 1.0	5.1 ± 1.0	0.12	4.8 ± 1.0	5.1 ± 1.2	0.07	5.1 ± 1.0	4.9 ± 1.0	0.33
HDL-Chol (mmol/l)	1.1 ± 0.3	1.2 ± 0.3	0.45	1.1 ± 0.3	1.1 ± 0.3	0.63	1.1 ± 0.3	1.2 ± 0.4	0.22
TG (mmol/l) #	1.6 (1.1 - 2.2)	1.4 (1.1 - 1.7)	0.24	1.6 (1.2 - 2.3)	1.5 (1.2 - 2.0)	0.37	1.6 (1.3 - 2.2)	1.6 (1.1 - 2.2)	0.20
25(OH)D (nmol/l) #	63 (37 - 90)	51 (33 - 93)	0.48	57.7 (40.3 - 79.2)	64.7 (40.1 - 91.6)	0.28	62.0 (35.2 - 87.9)	74.0 (39.2 - 96.1)	0.11
VDBP#	6.3 (04 - 54.5)	12 (7.8 - 37.3)	0.33	14.1 (5.2 - 63.2)	10.8 (5.9 - 85.4)	0.91	18.7 (6.0 - 46.6)	26.5 (5.7 - 70.1)	0.57
				**BMD**					
BMD Spine	1.2 ± 0.1	0.9 ± 0.1	<0.01	1.2 ± 0.2	0.8 ± 0.1	<0.01	1.1 ± 0.1	0.9 ± 0.1	<0.01
BMD DF left	1.0 ± 0.1	0.8 ± 0.1	<0.01	1.1 ± 0.1	0.8 ± 0.1	<0.01	1.0 ± 0.1	0.9 ± 0.1	<0.01
				**Bone Turnover markers**					
PTH (pg/ml) #	10 (7.4 - 15.7)	19 (11.3 - 27.5)	0.09	15.4 (8.2 - 33.7)	13.4 (7.0 - 31.4)	0.47	12.9 (6.7 - 21.7)	17.0 (9.6 - 40.2)	0.07
OPG (pg/ml) #	721 (474 - 954)	1065 (544 - 1285)	0.11	763 (597 - 989)	818 (623 - 1202)	0.36	752 (499 - 1137)	922 (520 - 1041)	0.35
OPN (pg/ml) #	2408 (1168 - 3127)	2902 (2398 - 4252)	0.15	2032 (796 - 3197)	2677 (1447 - 3726)	0.16	2382 (1446 - 3567)	2437 (1267 - 4427)	0.87
SOS T(pg/ml) #	832 (334 - 2197)	1889 (481 - 2295)	0.52	1354 (581 - 2112)	1339 (795 - 2446)	0.68	1538 (716 - 2430)	1694 (1028 - 2212)	0.56
FGF23(pg/ml) #	48 (43 - 83)	78 (44 - 86)	0.45	72 (44 - 85)	71 (41 - 80)	0.29	71 (42 - 81)	74 (44 - 84)	0.24
Osteocalcin(ng/ml) #	6 (2 - 12)	9 (3 - 14)	0.30	10 (3 - 15)	9 (5 - 14)	0.85	9 (4 - 13)	9 (2 - 13)	0.55

Note: Data presented as Mean ± SD for normal variables while Median (Q1 – Q3) for non-normal variables; # indicates non-normal variables. P-value < 0.05 considered significant; P-values are obtained from independent sample t-test and Mann-Whitney U test for normal and non-normal variables respectively. WHR: waist-hip ratio; BP: Blood pressure; Chol: cholestrol; TG: triglycerides; VDBP: vitamin D binding protein; DF: dual femur; PTH: parathyroid hormone; OPG: osteoprotegerin; OPN: osteopontin; SOST: sclerostin; FGF: fibroblast growth factor.

Supplementary Table S2. Association between *rs154440* genotypes and various other parameters

rs1544410	**TT**			**CT**			**CC**		
	Control	PMO	P-values	Control	PMO	P-values	Control	PMO	P-values
**General Characteristics**									
Age (Year)	53.3 ± 6.1	58.7 ± 8.4	<0.01	53.6 ± 5.7	57.1 ± 7.4	<0.01	54.5 ± 6.7	57.7 ± 8.4	<0.01
BMI (kg/m²)	34.5 ± 6.6	31.2 ± 6.9	0.01	34.2 ± 5.6	31.7 ± 6.1	<0.01	34.4 ± 6.0	31.2 ± 6.3	<0.01
Menarche (year) #	4.0 (2.0 - 6.5)	8.0 (4 - 14.0)	<0.01	4.0 (2.0 - 7.0)	9.0 (4 - 15.0)	<0.01	5.0 (3.0 - 8.0)	6.0 (4 - 15.0)	0.02
WHR	0.9 ± 0.1	0.9 ± 0.2	0.36	0.9 ± 0.1	0.9 ± 0.1	0.28	0.9 ± 0.1	0.9 ± 0.1	0.42
Systolic BP	125.7 ± 18.8	129.5 ± 19.8	0.29	126.7 ± 18.3	128.4 ± 20.0	0.50	127.2 ± 15.8	126.2 ± 16.3	0.69
Diastolic BP	76.6 ± 9.8	75.4 ± 10.3	0.51	75.7 ± 11.8	76.3 ± 11.4	0.66	76.9 ± 12.0	74.8 ± 9.7	0.22
Glucose (mmol/l)	7.8 ± 3.4	7.7 ± 3.3	0.83	8.1 ± 3.3	7.6 ± 3.3	0.20	7.3 ± 3.0	7.7 ± 3.2	0.38
Chol (mmol/l)	5.0 ± 0.9	4.9 ± 1.0	0.82	5.0 ± 1.0	5.1 ± 1.1	0.48	4.9 ± 1.1	5.1 ± 1.1	0.19
HDL-Chol (mmol/l)	1.1 ± 0.4	1.2 ± 0.4	0.14	1.1 ± 0.3	1.2 ± 0.4	0.85	1.1 ± 0.3	1.1 ± 0.3	0.48
TG (mmol/l) #	1.6 (1.3 - 2.3)	1.6 (1.1 - 2.1)	0.50	1.6 (1.3 - 2.3)	1.5 (1.2 - 2.0)	0.06	1.6 (1.1 - 2.2)	1.5 (1.2 - 2.0)	0.66
25(OH)D (nmol/l) #	65.3 (33 - 92)	59.6 (39 - 89)	0.57	57.8 (37 - 81)	70.5 (40 - 93)	0.07	62.6 (46 - 83)	59.4 (34 - 96)	0.62
VDBP#	8.5 (6 - 40)	45.3 (9 - 100)	0.08	31.7 (6 - 100)	10.7 (5 - 54.7)	0.40	10.1 (4 - 53.8)	11.9 (6 - 69.9)	0.28
**BMD**									
BMD Spine	1.1 ± 0.1	0.9 ± 0.1	<0.01	1.1 ± 0.2	0.8 ± 0.1	<0.01	1.2 ± 0.1	0.9 ± 0.1	<0.01
BMD DF left	1.0 ± 0.1	0.9 ± 0.1	<0.01	1.0 ± 0.1	0.8 ± 0.1	<0.01	1.0 ± 0.1	0.8 ± 0.1	<0.01
BMD DF Right	1.0 ± 0.1	0.8 ± 0.1	<0.01	1.0 ± 0.1	0.8 ± 0.1	<0.01	1.0 ± 0.1	0.8 ± 0.1	<0.01
**Bone Turnover markers**									
PTH (pg/ml) #	12.6 (9 - 20.4)	16.8 (9 - 39.2)	0.26	14.0 (7 - 21.8)	16.0 (8 - 42.8)	0.55	11.1 (7 - 20.4)	14.1 (8 - 27.5)	0.28
OPG (pg/ml) #	802 (605- 1138)	922(641 - 1163)	0.42	746 (598 - 926)	843 (623-1072)	0.10	684 (480 - 996)	793 (558- 1202)	0.31
OPN (pg/ml) #	2277 (1446-3929)	2515 (1242-4544)	0.89	2339 (888 - 3587)	2687 (1708- 3755)	0.54	2311 (946-2916)	2786 (1398 - 3821)	0.11
SOST (pg/ml) #	1818 (845 - 2635)	1696 (895 - 3008)	0.79	1519 (800 - 2825)	1693 (1016-2442)	0.84	906.4 (373- 1550)	1254 (594 - 2040)	0.15
FGF23 (pg/ml) #	52.7 (39.5 - 85.6)	72.9 (43.8 - 81.1)	0.56	73.6 (43.6 - 84.6)	74.7 (43.8 - 82.4)	0.71	60.7 (42.9 - 79.3)	71.9 (41.2 - 81.9)	0.85
Osteocalcin (ng/ml) #	9.6 (7.3 - 13.1)	8.2 (3.6 - 12.9)	0.20	7.7 (2.3 - 13.1)	9.9 (4.8 - 13.9)	0.16	8.2 (3.1 - 13.6)	8.0 (3.0 - 14.7)	0.81

Note: Data presented as Mean ± SD for normal variables while Median (Q1 – Q3) for non-normal variables; # indicates non-normal variables. P-value < 0.05 considered significant; P-values are obtained from independent sample t-test and Mann-Whitney U test for normal and non-normal variables respectively. WHR: waist-hip ratio; BP: Blood pressure; Chol: cholestrol; TG: triglycerides; VDBP: vitamin D binding protein; DF: dual femur; PTH: parathyroid hormone; OPG: osteoprotegerin; OPN: osteopontin; SOST: sclerostin; FGF: fibroblast growth factor.
